# Motor network reorganization after motor imagery training in stroke patients with moderate to severe upper limb impairment

**DOI:** 10.1111/cns.14065

**Published:** 2022-12-27

**Authors:** Hewei Wang, Xin Xiong, Kexu Zhang, Xu Wang, Changhui Sun, Bing Zhu, Yiming Xu, Mingxia Fan, Shanbao Tong, Xiaoli Guo, Limin Sun

**Affiliations:** ^1^ Department of Rehabilitation Medicine Huashan Hospital Fudan University Shanghai China; ^2^ School of Biomedical Engineering Shanghai Jiaotong University Shanghai China; ^3^ Shanghai Key Laboratory of Magnetic Resonance East China Normal University Shanghai China

**Keywords:** functional connectivity, motor imagery training, stroke rehabilitation, task‐based fMRI, upper limb function

## Abstract

**Background:**

Motor imagery training (MIT) has been widely used to improve hemiplegic upper limb function in stroke rehabilitation. The effectiveness of MIT is associated with the functional neuroplasticity of the motor network. Currently, brain activation and connectivity changes related to the motor recovery process after MIT are not well understood.

Aim: We aimed to investigate the neural mechanisms of MIT in stroke rehabilitation through a longitudinal intervention study design with task‐based functional magnetic resonance imaging (fMRI) analysis.

**Methods:**

We recruited 39 stroke patients with moderate to severe upper limb motor impairment and randomly assigned them to either the MIT or control groups. Patients in the MIT group received 4 weeks of MIT therapy plus conventional rehabilitation, while the control group only received conventional rehabilitation. The assessment of Fugl‐Meyer Upper Limb Scale (FM‐UL) and Barthel Index (BI), and fMRI scanning using a passive hand movement task were conducted on all patients before and after treatment. The changes in brain activation and functional connectivity (FC) were analyzed. Pearson's correlation analysis was conducted to evaluate the association between neural functional changes and motor improvement.

**Results:**

The MIT group achieved higher improvements in FM‐UL and BI relative to the control group after the treatment. Passive movement of the affected hand evoked an abnormal bilateral activation pattern in both groups before intervention. A significant *Group* × *Time* interaction was found in the contralesional S1 and ipsilesional M1, showing a decrease of activation after intervention specifically in the MIT group, which was negatively correlated with the FM‐UL improvement. FC analysis of the ipsilesional M1 displayed the motor network reorganization within the ipsilesional hemisphere, which correlated with the motor score changes.

**Conclusions:**

MIT could help decrease the compensatory activation at both hemispheres and reshape the FC within the ipsilesional hemisphere along with functional recovery in stroke patients.

## Graphical abstract

The neural mechanism of motor imagery training in stroke patients with moderate to severe upper limb motor impairments was investigated using task‐based fMRI. The results revealed that motor imagery training during motor recovery decreased overaction at both hemispheres and reorganized the motor network within the ipsilesional hemisphere.

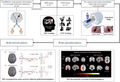

## 1 INTRODUCTION

Stroke represents a major cause of long‐term adult disability worldwide.^1^ More than 50% of survivors still suffer from varying degrees of upper limb motor impairment half a year after stroke.^2^, ^3^ In clinical practice, repeated, task‐specific, and active training is critical to effective post‐stroke upper limb motor function rehabilitation.^4^ However, active movement training, such as constraint‐induced movement therapy, relies heavily on the residual functioning of the patients, limiting its application in patients with poor motor performance.^5^, ^6^

Mental practice of movements, also known as motor imagery training (MIT), has attracted much interest for its potential for neurorehabilitation to improve upper limb function.^7^, ^8^ Unlike active movement training, MIT involves the cognitive rehearsal of specific actions without overt motor output. It is, therefore, applicable for stroke patients with severe motor deficits^9^, ^10^ due to their reserved motor imagery abilities.^11^ Sharma et al.^7^ suggested MIT as an intriguing new “backdoor” approach to accessing the motor system at all stages of stroke recovery, especially for patients with severe motor impairment. Their suggestion is supported by ample evidence that imagery of movements and actual motor output have similar neural substrates in healthy subjects^12^, ^13^ and stroke patients.^13^, ^14^ To date, MIT has been widely used in clinical practice. Clinical guidelines and randomized controlled studies have highlighted its effectiveness in post‐stroke upper limb function rehabilitation.^4^, ^15^, ^16^

Neural plasticity forms the intrinsic basis of neurorehabilitation for modern rehabilitation medicine and has been applied in restoring motor functions after stroke. Recently, motor task‐based neuroimaging studies have demonstrated that motor relearning is accompanied by reorganizing of motor networks.^17^ As an effective intervention for motor rehabilitation, MIT is essential in motor network reorganization. For example, after MIT, stroke patients showed more focal activation in the ipsilesional primary motor cortex (M1)^18^ and increased activation in the bilateral premotor cortex and M1^19^ when executing movements of the affected hand. Additionally, brain activation of stroke patients after MIT increased in the ipsilesional primary somatosensory cortex and was attenuated in the contralesional M1 when performing the corresponding motor imagery tasks.^20^ Moreover, the effective connectivity in the motor executive network was enhanced during either motor execution or motor imagery tasks after MIT.^21^ However, none of these studies targeted patients with severe upper limb motor impairment due to their difficulty in conducting motor execution tasks. As a result, exploring the neural substrates of MIT in the majority of its target patient population is challenging, given that the applicability in severe patients is an essential advantage of MIT.

Passive movements are an alternative to the task of active movements. An fMRI study showed that an active or passive palm‐finger brushing task produced broadly equivalent brain activation in the sensorimotor areas in healthy adults.^22^ A comparative study showed no difference in brain activation patterns when healthy subjects completed identical active and passive hand opening/closing tasks.^23^ In stroke patients, passive movement closely resembled the pattern of brain activation in overt execution.^24^, ^25^ An activation likelihood estimation meta‐analysis demonstrated that both active and passive tasks induced convergent activation of M1 and supplementary motor area (SMA).^26^ These consistent findings support using passive movements as an alternative in hemiplegic patients who cannot perform required movement tasks. Moreover, the fMRI tasks of passive movements have shown high reliability in test–retest reproducibility studies in healthy people and stroke patients.^27^, ^28^

In addition to the activation analysis, the functional connectivity (FC) analysis may provide more information about the mechanism underlying the treatment effects of MIT at the brain network level. Simple task‐related brain activity is frequently more intensive and with a broader range of activation in stroke patients than in healthy controls, while more complex tasks usually involve lower levels of cortical activity.^29^ The observed phenomenon has been associated with disturbed sensorimotor integrative networks of the brain affected by stroke lesions; the former derives from compensatory activation, while the latter is probably related to the need for more connections between integrative sensorimotor regions that are dysfunctional after stroke.^30^ Unlike most task‐based fMRI studies focusing on local brain activation, FC analysis of task‐based fMRI data allows for the assessment of relationships of brain activities across brain areas.^31^

To deepen our understanding of the neural mechanism of MIT in stroke patients with poor motor function, we recruited patients with moderate to severe upper limb impairment and adopted a passive hand movement task for fMRI scanning. We hypothesized that in this major target patient population, the efficacy of MIT is associated with specific reorganizations in the motor network, which we aimed to reveal in this study from brain activation and seed‐based FC analyses.

## 2 METHODS

### 2.1 Patients and interventions

**FIGURE 1:**
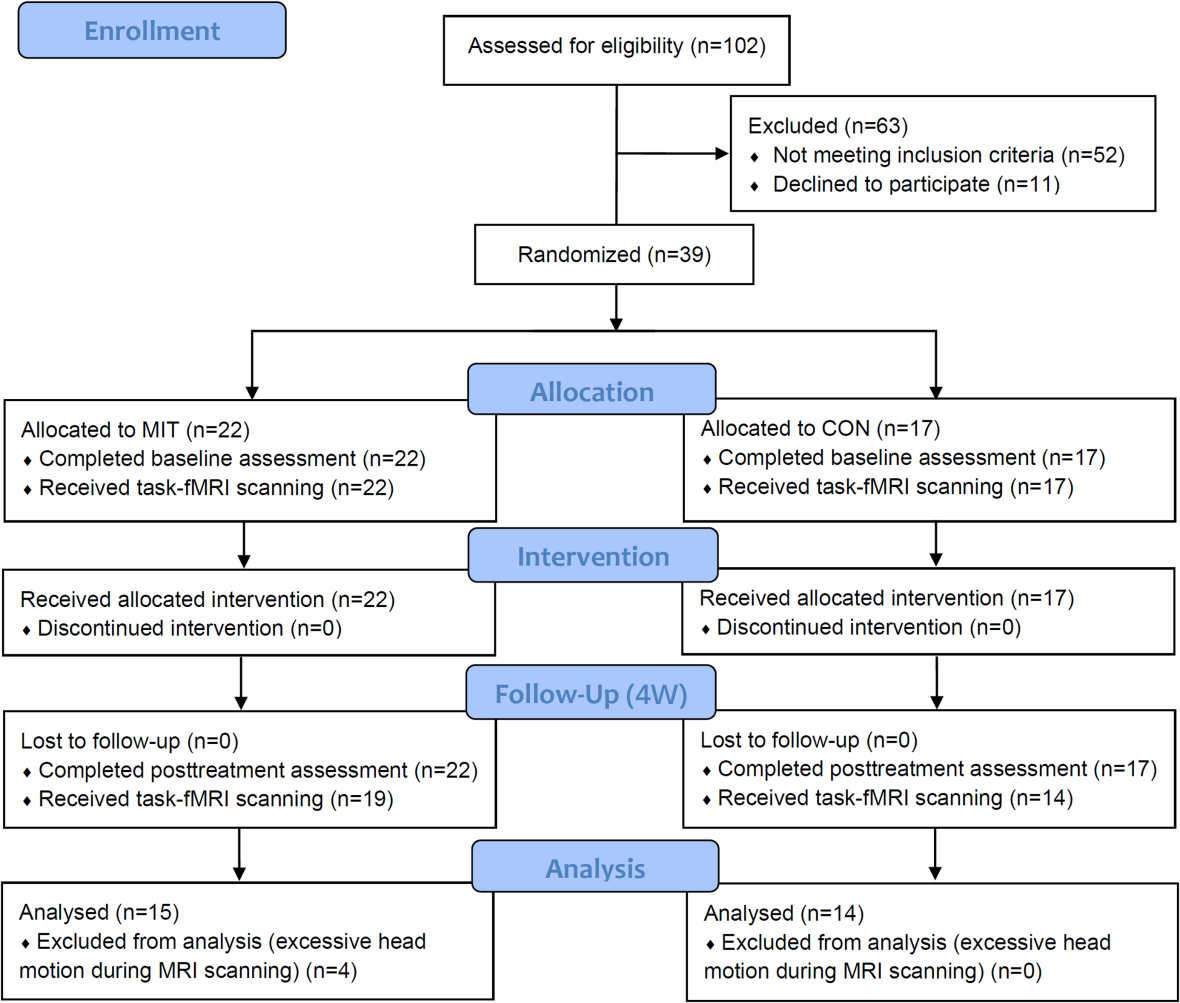
CONSORT flow diagram of the study design

This study was a randomized, single‐blind, controlled trial conducted at Huashan Hospital in Shanghai, China. All potential participants were screened by the physicians for eligibility. Each participant signed a written informed consent before participation. This study was approved by the Review Board of Ethics Committee of Huashan Hospital and registered at the Chinese Clinical Trial Registry (ChiCTR‐TRC‐08003005). The study design and protocol are shown in a flow diagram according to CONSORT guidelines (Figure. 1). A total of thirty‐nine stroke patients were recruited from the inpatient services based on previously established eligibility criteria.^9^

Inclusion criteria: (1) first‐ever stroke (infarct or hemorrhage); (2) stroke onset between 3 and 12 months before enrollment for the study; (3) no significant cognitive impairment (Mini‐Mental State Examination ≥ 27); (4) age between 18 and 80 years; (5) unilateral upper limb and hand hemiplegia (Brunnstrom stage ≤ grade IV); and (6) right‐handed before stroke according to the Edinburgh Handedness scale.

Exclusion criteria: (1) severe spasticity (Modified Ashworth Spasticity Scale > 2) of the affected upper extremity; (2) significant pain on the affected side (Ten‐point Visual Analog Scale > 4); (3) excessive sensory disturbance, aphasia, neglect, or apraxia; (4) active malignant disease or multiple organ failure; (5) presently enrolled in any other rehabilitation or drug studies.

The sample size was calculated before the start of the study using the stepped rules of thumb for pilot trials.^32^ The effect size was approximately 0.5 in the Upper‐Extremity Fugl‐Meyer score for the main trial, as speculated from our previous studies and other related studies in this field.^9^, ^10^, ^20^, ^33^ Furthermore, a dropout of 10% was assumed, and the final target sample size was 34 (17 per group) when setting the planned main trial with a power of 90% and a type I error rate of 5%.

All recruited patients were randomly assigned to one of the two groups, that is, the control group (CON group, *n* = 17), in which patients only received conventional rehabilitation therapy, and the motor imagery training group (MIT group, *n* = 22) in which patients received motor imagery training in addition to conventional rehabilitation. The randomization procedure was performed by an independent physician who was not involved in the recruitment, intervention, and assessment of the participants. Computer‐generated random numbers were concealed in sequentially numbered opaque envelopes to ensure allocation randomness. Therapists who delivered the conventional rehabilitation therapy and the recruited patients were blinded to study allocation.

All patients underwent rehabilitation interventions 5 days a week for 4 weeks. Daily interventions included 3 h of conventional rehabilitation therapy and 30 min of group‐specific treatments; motor imagery training for the MIT group and health education on stroke information or patient–physician consultation for the CON group.^33^ An experienced physiotherapist supervised the MIT training in a quiet treatment room. The MIT consisted of: (1) the patients imagined themselves in a relaxing environment for 3 min; (2) the patients practiced motor imagery of basic movements such as opening and closing the hand, elevating the arm, and extending the elbow, for 10 min; (3) imagery of progressive task‐oriented training or activities of daily living such as reaching and grasping, doing laundry, and using a hairbrush or comb for 15 min; (4) 3 min of refocusing on the room. We adopted first‐person MIT to the affected upper extremity during training. Patients engaged in MIT training through standardized verbal instruction from the therapist to ensure full engagement. The therapist adopted individualized training tasks for each patient. Patients were intermittently checked to assess their ability to imagine and vividly experience the motor tasks.

All the patients were assessed for upper extremity motor function and activities of daily living by an independent physician blinded to the treatment condition, before and after the 4‐week interventions. The primary outcome measure was the Upper Extremity Fugl‐Meyer score which is a valid and reliable tool for evaluating upper limb motor level within and between raters in stroke survivors,^34^ consisting of 33 items scored on a three‐point (0, 1, 2) ordinal scale.^35^ The secondary outcome measure was the Barthel Index (BI), a 10‐item scale that evaluates the independence of a patient in conducting basic daily living activities with a maximum possible score of 100. Research indicates that BI is an appropriate outcome measure for stroke trials and clinical practice because of its excellent inter‐rater reliability.^36^

### 2.2 fMRI data acquisition

T1‐ and T2‐weighted structural and task‐based functional images were acquired on a 3 T MRI scanner (Siemens) at Shanghai Key Laboratory of Magnetic Resonance. T1‐weighted images were obtained using a magnetization‐prepared rapid gradient echo sequence (TR = 1900 ms, TE = 3.42 ms, TI = 900 ms, FOV = 240 × 240 mm^2^, flip angle = 9°, imaging matrix = 256 × 256, 192 sagittal slices, thickness = 1 mm, gap = 0.5 mm). T2‐weighted images were obtained using a transverse turbo‐spin‐echo sequence (TR = 6000 ms, TE = 93 ms, FOV = 220 × 220 mm^2^, flip angle = 120°, imaging matrix = 320 × 320, 30 axial slices, thickness = 5 mm, no gap) for lesion identification. Functional images were acquired using an echo‐planar imaging sequence (TR = 3000 ms, TE = 30 ms, FOV = 220 × 220 mm^2^, flip angle = 90°, imaging matrix = 64 × 64, 30 axial slices, 5 mm thickness, no gap). During MRI scanning, subjects were instructed to remain awake but motionless with their eyes closed.

**FIGURE 2:**
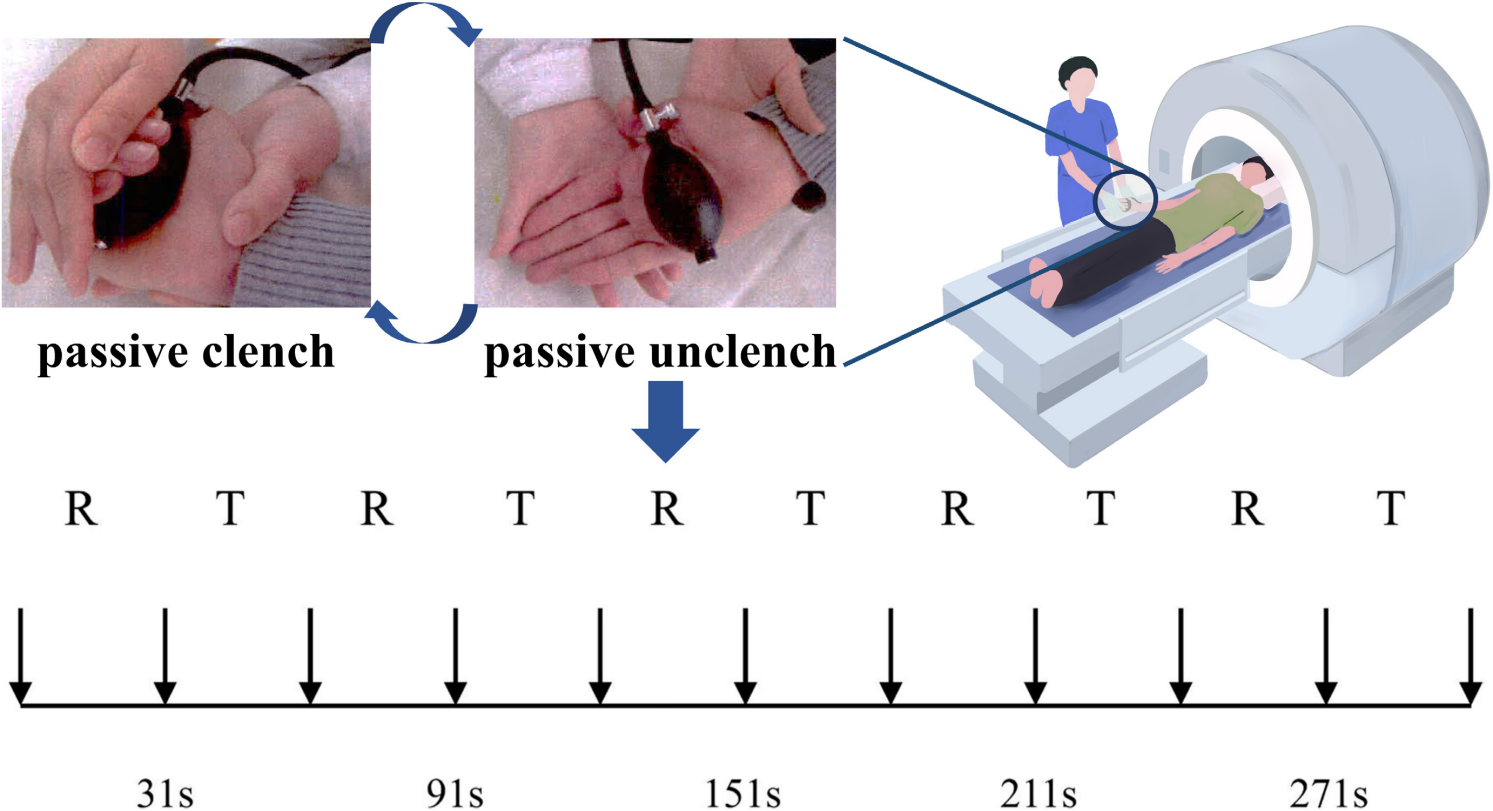
Block design for task‐based fMRI. The patients clutched their hands passively at 1 Hz with the help of an experimenter. An air‐filled rubber bulb was placed in the patient's hand to maintain consistency in movement amplitude. A total of 10 blocks including 5 REST and 5 TASK were adopted for the tb‐based fMRI. Each block spanned for 30 s. R, REST; T, TASK

The fMRI scanning included two sessions, each with the affected and unaffected hands performing passive finger flexion–extension tasks. A 6 s dummy scan was performed at the start of each session to stabilize the BOLD signal, which was then discarded in the subsequent analysis. A block design was then adopted with 5 rest blocks and 5 task blocks in each session (10 volumes/30 s for each block). During each task block, passive movements were conducted with an experimenter who was blinded to the treatment condition. The experimenter was instructed to clench the hands of the subject into a fist following a voice prompt at 1 Hz under stable movement amplitude (Figure 2). Before the formal experiment, subjects were asked to practice and adapt to the passive movements. Thirty‐three patients (19 in the MIT group and 14 in the CON group) finished two sessions of task‐fMRI scanning before and after the intervention (Table 1). No mirror movements or synkinesia were observed in all patients during scanning.

**TABLE 1:** Demographic and clinical information for the stroke patients

Group‐ID	Gender	Age (years)	Side of lesion	Site of lesion	Stroke type	Days after stroke	Lesion volume (cm^3^)	FM‐UL‐1	FM‐UL‐2	Included in task‐fMRI analyses
MIT‐1	M	63	R	BG, CO	I	97	6.15	10	24	Yes
MIT‐2	M	61	L	BG, CR	I	97	38.01	9	26	Yes
MIT‐3	M	61	L	BG, CR	I	115	4.77	10	29	Yes
MIT‐4	M	65	L	BG	H	106	5.37	26	48	Yes
MIT‐5	M	63	L	BG	I	142	1.92	36	46	Yes
MIT‐6	M	32	L	BG	H	126	7.61	14	21	No
MIT‐7	M	52	L	BG	H	220	6.83	9	18	Yes
MIT‐8	M	27	L	BG	H	89	6.58	9	30	No
MIT‐9	M	62	L	BG	H	147	10.14	9	24	No
MIT‐10	M	25	R	BG	H	92	22.12	15	35	Yes
MIT‐11	M	52	R	BG	H	100	27.96	30	58	Yes
MIT‐12	M	57	R	BG	H	89	18.79	7	17	Yes
MIT‐13	M	58	R	BG	H	90	19.35	4	8	No
MIT‐14	F	63	R	BG, CR	I	145	5.12	8	14	Yes
MIT‐15	M	65	R	Pons	I	154	20.27	39	47	Yes
MIT‐16	M	56	L	BG	I	108	51.83	5	21	Yes
MIT‐17	M	55	L	BG, CR	I	130	1.33	17	38	Yes
MIT‐18	M	68	R	BG	H	87	18.33	30	54	Yes
MIT‐19	M	45	L	BG	H	95	37.25	10	29	Yes
MIT‐20	F	60	R	Brainstem	I	162	0.80	35	40	No
MIT‐21	M	63	L	Tha	H	138	6.47	24	42	No
MIT‐22	M	43	R	BG	H	178	5.66	12	26	No
CON‐1	M	55	R	CR	I	145	3.00	27	48	Yes
CON‐2	M	58	L	BG	H	265	28.59	4	5	Yes
CON‐3	M	69	L	BG	H	119	2.70	6	7	Yes
CON‐4	M	61	L	BG, CO, Pons	H	102	12.85	8	11	Yes
CON‐5	M	65	L	CR	I	143	3.62	38	42	Yes
CON‐6	M	59	L	CR, BG	I	114	5.29	16	20	Yes
CON‐7	M	70	R	BG, CR	H	162	9.74	6	7	Yes
CON‐8	M	62	R	BG	H	92	16.47	33	60	Yes
CON‐9	M	56	R	BG, CR	I	172	1.45	38	62	Yes
CON‐10	M	62	L	BG	I	136	31.08	27	33	Yes
CON‐11	M	55	R	BG	H	149	39.60	8	10	No
CON‐12	M	64	R	CR, BG	I	182	20.24	16	16	Yes
CON‐13	M	69	L	BG	I	303	6.24	32	32	No
CON‐14	F	59	R	BG	I	93	33.92	7	7	No
CON‐15	M	61	R	BG, IC, CR	I	91	7.21	15	20	Yes
CON‐16	M	61	L	BG, CO	H	138	22.80	4	6	Yes
CON‐17	M	29	R	BG	H	124	2.81	1	3	Yes
*p*	0.709 (*χ* ^*2*^)	0.124 (*U*)	0.643 (*χ* ^*2*^)	/	0.455 (*χ* ^*2*^)	0.131 (*U*)	1.000 (*U*)	0.644 (*U*)	0.052 (*U*)	/

Abbreviations: BG, basal ganglia; CO, centrum ovale; CON, control group; CR, corona radiate; F, female; FM‐UL‐1, Fugl‐Meyer Assessment Upper Limb subscale before intervention; FM‐UL‐2, Fugl‐Meyer Assessment Upper Limb subscale after the intervention; H, hemorrhage; I, ischemia; IC, internal capsule; L, left; M, male; MIT, motor imagery training group; R, right; Tha, thalamus.

### 2.3 fMRI preprocessing

Images of the patients with left‐sided lesions were flipped before preprocessing. After flipping, the right hemisphere was tagged as the ipsilesional side and the left hemisphere as the contralesional side. fMRI preprocessing was carried out using DPARSF,^37^ which provides a pipeline workspace based on Statistical Parametric Mapping (SPM, Wellcome Trust Centre for Neuroimaging, London). First, volumes from each run were realigned to their first volume to estimate motion artifacts. The head motion was then corrected using a six‐parameter rigid body spatial transformation. Functional mean images were obtained and co‐registered to the corresponding individual structural image before normalization to the standard Montreal Neurological Institute (MNI) template. Finally, images were smoothed with an 8‐mm isotropic Gaussian kernel. Four patients (in the MIT group) with excessive head motion during MRI scanning were excluded from the task‐fMRI analyses.

### 2.4 Activation and FC analysis

A general linear model (GLM) was set for each individual in SPM first‐level statistical analysis to estimate the task‐related activation by contrasting passive movement and rest conditions (Passive movement > Rest). In this model, head motion in the six directions estimated in the preprocessing was added as a regressor of no interest.

In addition to activation analysis, seed‐based FC analysis was performed with the CONN toolbox.^38^ Clusters with significantly different activation alterations between the two groups and correlation with FM‐UL improvement (detailed coordination was listed in the results) were selected as seeds. Pearson's correlation coefficient between voxel and seeds was used to calculate the FC.

### 2.5 Statistical analysis

The normality of variables (age, days after stroke onset, lesion volume, FM‐UL, and BI score) was tested by Shapiro–Wilk's test, showing that none of these variables conform to the normal distribution. Group differences were tested by chi‐square tests on sex, stroke type, and lesion side, and Mann–Whitney *U*‐test on age, days after stroke onset, lesion volume, FM‐UL, and BI score. A robust repeated‐measures analysis of variance (ANOVA) was performed on FM‐UL and BI to verify the efficacy of MIT, taking *Group* (2 levels: MIT and CON) as the between‐subject factor and *Time* (2 levels: before and after intervention) as the within‐subject factor. Wilcoxon signed‐ranked test was performed to compare FM‐UL before and after the intervention in the post hoc analysis.

Voxel‐wise 2 × 2 (*Group* × *Time*) repeated ANOVAs and voxel‐wise correlation analyses with FM‐UL improvement were performed on activation/FC using FSL Randomize (www.fmrib.ox.ac.uk/fsl/randomise). The statistical map was corrected by threshold‐free cluster enhancement (TFCE corrected *p* < 0.05, H = 2, E = 0.5, number of permutations = 5000) with a cerebral gray matter mask to control for the family‐wise error. Brain regions with different alterations in activation/FC between the two groups were detected with significant *Group* × *Time* interaction. The activation/FC values from the clusters with significant *Group* × *Time* interaction and correlation with FM‐UL improvement were extracted and averaged for post hoc analysis.

## 3 RESULTS

### 3.1 Clinical outcome

**FIGURE 3:**
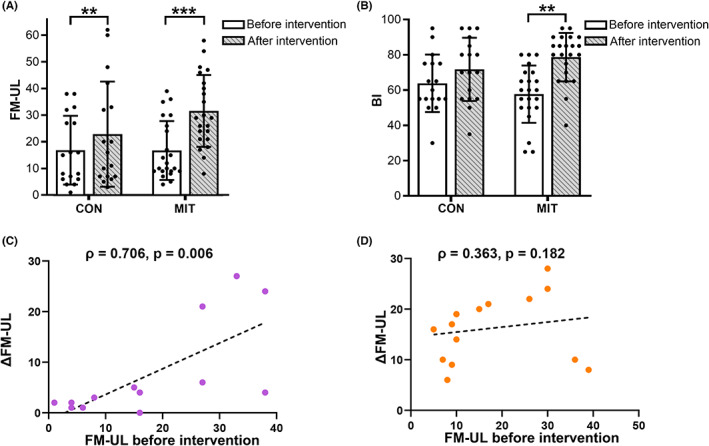
Improvement in FM‐UL score after rehabilitation in the motor imagery training group (MIT) and control group (CON). (A) FM‐UL score before and after rehabilitation in the two groups; (B) Comparison of FM‐UL improvement between the two groups; (C, D) Correlations between FM‐UL improvement (ΔFM‐UL) and FM‐UL score before intervention in the CON (C) and MIT (D) groups. Error bars indicate standard deviation, **p < 0.01, ***p < 0.001. CON, control group; M‐UL, Fugl‐Meyer Assessment Upper Limb; MIT, motor imagery training group

Detailed demographic and clinical data for the patients are shown in Table 1. There were no significant differences in age, sex, stroke type, lesion side, lesion volume, days after stroke onset, FM‐UL, and BI between the two groups before intervention (all *p* > 0.05, Table 1). Both groups exhibited a significant improvement in FM‐UL (MIT before and after intervention: 16.73 ± 11.06 vs. 31.59 ± 13.50, *p* < 0.001; CON before and after intervention: 16.82 ± 12.91 vs. 22.88 ± 19.71, *p* = 0.001, Figure 3A). Only MIT group demonstrated significant improvements in BI after interventions (MIT before and after intervention: 57.73 ± 16.24 vs. 78.64 ± 13.73, *p* < 0.001; CON before and after intervention: 63.82 ± 16.25 vs. 71.71 ± 17.94, *p* = 0.160, Figure 3B). Robust repeated‐measures ANOVA showed significant Group × Time interaction for FM‐UL (*F*_1,36_ = 35.27, *p* < 0.001) and BI (*F*_1,36_ = 8.79, *p* = 0.007). FM‐UL improvement was greater in the MIT group than in the CON group (MIT: 14.86 ± 6.71, CON: 6.06 ± 8.80, *p* < 0.001), demonstrating adjunctive treatment effects of motor imagery training.

To investigate the dependency of the treatment efficacy of motor imagery training on preserved motor ability, we analyzed the correlation between the improvement in FM‐UL score and the baseline FM‐UL score before rehabilitation. Spearman's correlation revealed a significant correlation in the CON group (*ρ* = 0.706, *p* = 0.006, Figure 3C) and not in the MIT group (*ρ* = 0.363, *p* = 0.182, Figure 3D). These results suggest that conventional rehabilitation therapy is less effective in patients with severe stroke. In contrast, the efficacy of motor imagery training is less dependent on reserved motor ability.

### 3.2 Brain activation to passive movements

Twenty‐nine patients (15 in the MIT group and 14 in the CON group) who had completed task‐fMRI scanning before and after intervention and with good image quality were included in the fMRI analysis.

**FIGURE 4:**
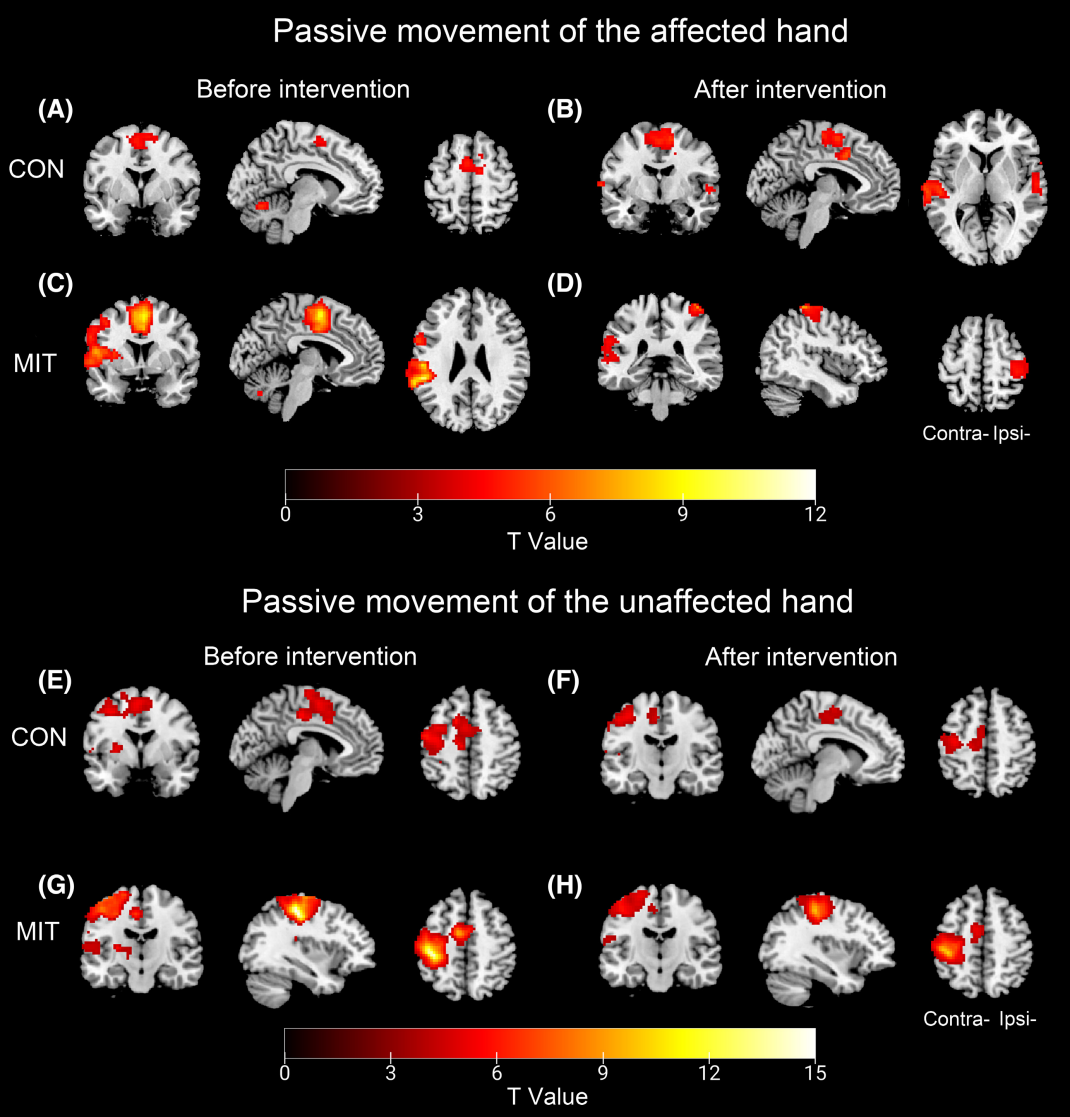
Brain activation in response to hand passive movements. (A‐D) Brain activation in response to passive movement of the unaffected hand before and after intervention in the CON group and MIT group; (E‐H) Brain activation in response to passive movement of the affected hand before and after intervention in the CON and MIT groups. All clusters are significant at voxel‐level uncorrected *p* < 0.001 and cluster size ≥ 100 voxels). CON, control group; Contra‐, contralesional side; Ipsi‐, ipsilesional side; MIT, motor imagery training group.

The general activation to passive movements of the affected/unaffected hand is shown in Figure 4 and Table S1 (voxel‐level uncorrected *p* < 0.001 and cluster size ≥ 100). During passive movement of the unaffected hand, both groups showed a typical contralateral‐lateralized activation pattern before and after the intervention, with activation mainly in the contralesional precentral gyrus (M1), postcentral gyrus (S1), SMA, and the ipsilesional cerebellum. On the other hand, the activation to passive movement of the affected hand before intervention showed an abnormal bilateral activation pattern, which focused on the bilateral SMA and cerebellum.

**FIGURE 5:**
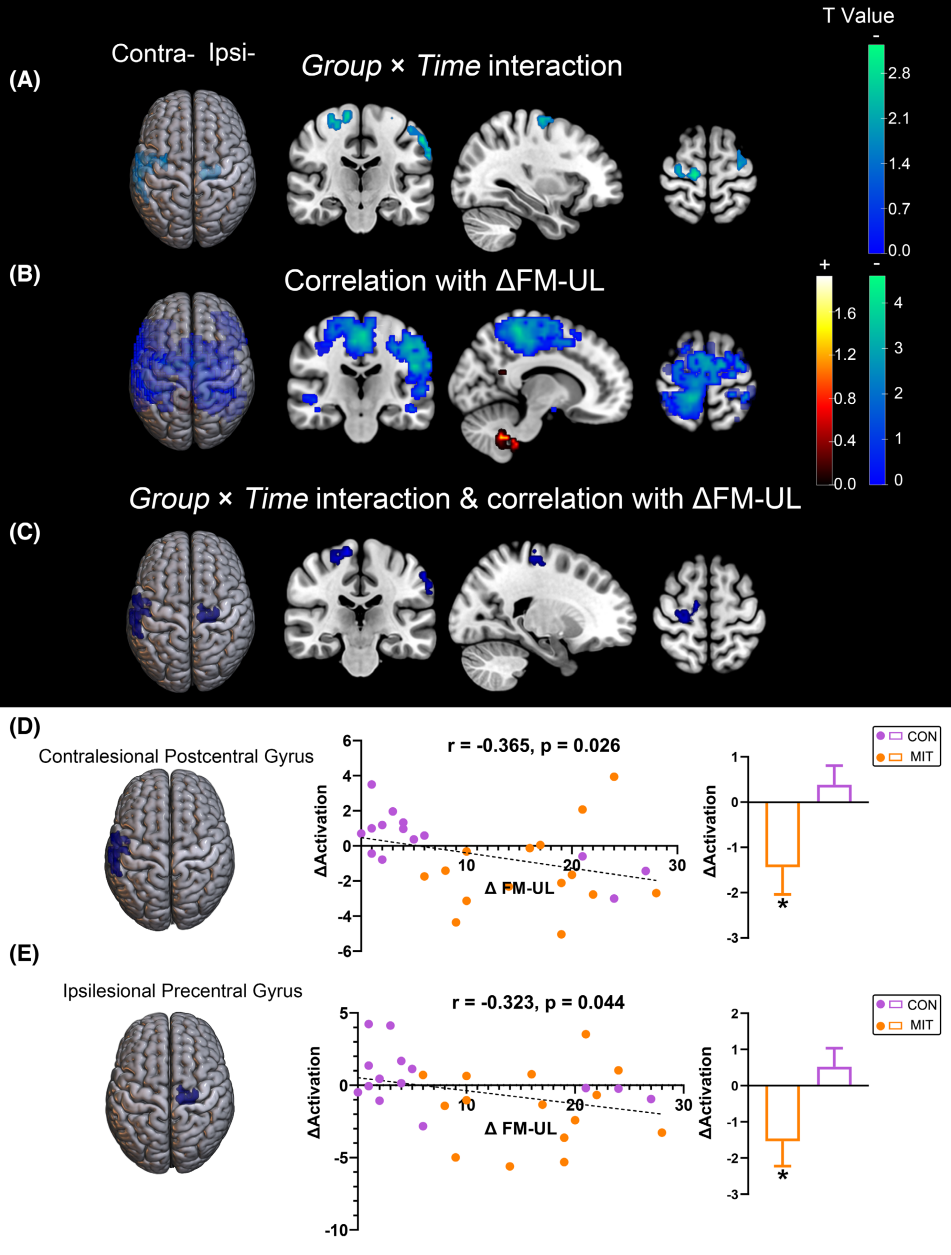
Alterations in brain activation in response to passive movement of the affected hand between the MIT and CON groups. (A) Brain regions in which activation exhibited significant Group × Time interactions (threshold‐free cluster enhancement [TFCE] corrected *p* < 0.05 and cluster size ≥ 100 voxels). A positive T value indicates that activation was increased in the MIT group but decreased in the CON group after the intervention. A negative T value indicates that activation was decreased in the MIT group but increased in the CON group after intervention. Only regions with negative T values were found. (B) Brain regions in which activation change significantly correlates with FM‐UL improvement (TFCE corrected *p* < 0.05 and cluster size ≥ 100 voxels). A positive T value represents a positive correlation, and a negative T value represents a negative correlation. (C‐F) Brain regions in which activation showed significant Groups × Time interactions and correlations with FM‐UL improvement. Significant clusters are shown in (D‐E). For each cluster, the changes in activation (Δ Activation) are correlated with FM‐UL improvement (ΔFM‐UL) and compared between groups. *p < 0.05 compared between before and after the intervention.

ANOVA and correlation analysis results are shown in Figure 5 and Table S2. ANOVA analysis of activation demonstrated that the additional use of motor imagery training had no significant effects (*Group* × *Time* interaction) on the passive movement of the unaffected hand. On the other hand, passive movement of the affected hand showed significant *Group* × *Time* interaction, and post hoc analysis revealed that activations in the contralesional S1 (cluster size = 184, peak MNI: −59, −29, 47) and the ipsilesional M1 (cluster size = 100, peak MNI: 12, −24, 69) decreased significantly in the MIT group after intervention (contralesional S1: *p* = 0.031; ipsilesional M1: *p* = 0.043), compared with the CON group (both *p* > 0.32). Furthermore, correlation analyses revealed that the activation changes in these two areas were significantly negatively correlated with FM‐UL improvement (contralesional S1: *r* = −0.365, *p* = 0.026; ipsilesional M1: *r* = −0.323, *p* = 0.044).

### 3.3 Functional connectivity

**FIGURE 6:**
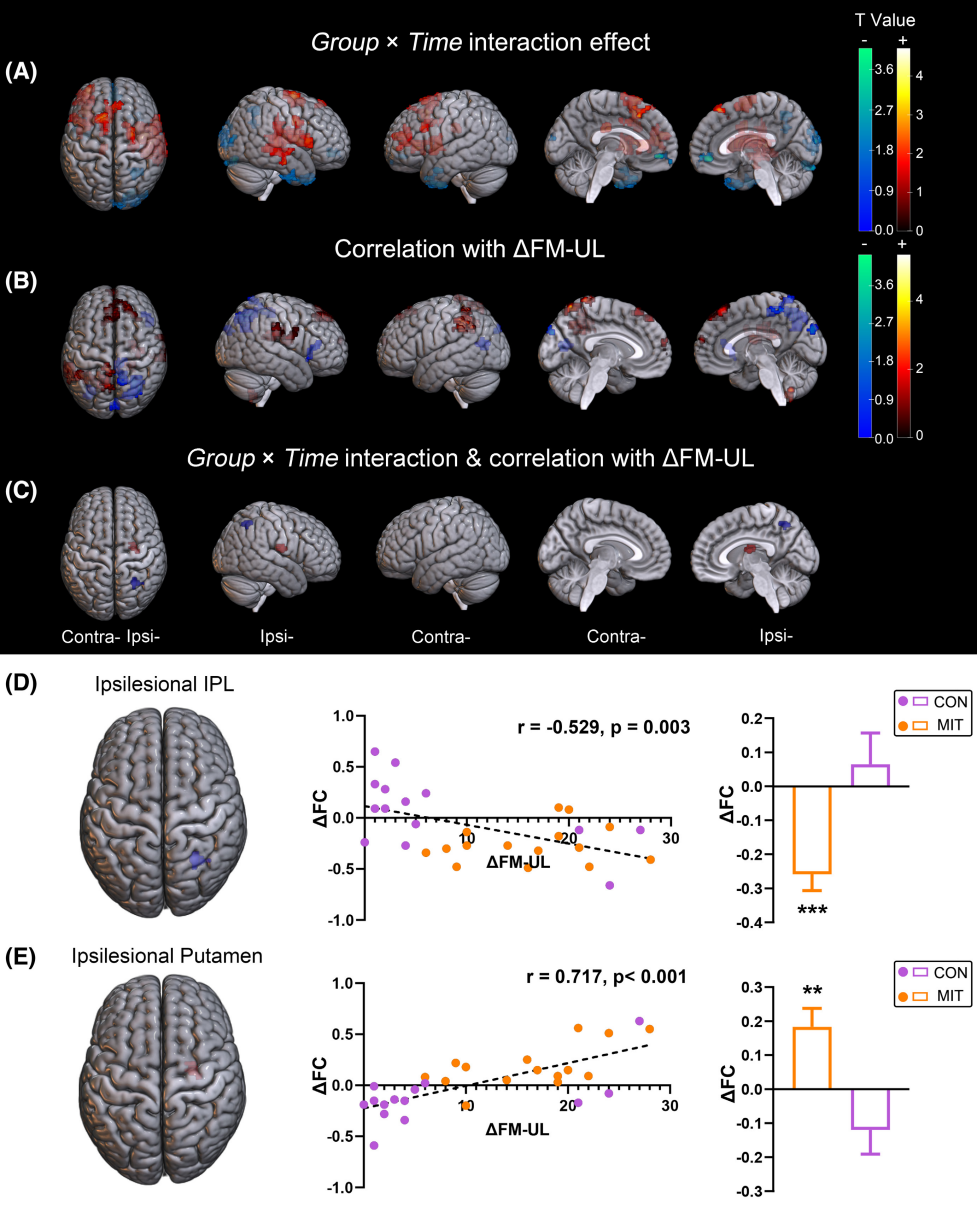
Changes in functional connectivity (FC) with the ipsilesional precentral cluster between MIT and CON groups. (A) Brain regions in which FC showed significant Group × Time interactions (threshold‐free cluster enhancement [TFCE] corrected *p* < 0.05 and cluster size ≥ 100 voxels). A positive T value indicates that FC was increased in the MIT group but decreased in the CON group after the intervention. A negative T value indicates that FC was decreased in the MIT group but increased in the CON group after intervention. (B) Brain regions in which FC was significantly correlated with FM‐UL improvement (TFCE corrected *p* < 0.05 and cluster size ≥ 100 voxels). A positive T value indicates a positive correlation, and a negative T value indicates a negative correlation. (C) Brain regions in which FC showed significant Group × Time interactions and was correlated with FM‐UL improvement. (D‐E) Post hoc analysis of significant Group × Time interactions and correlations with FM‐UL improvement on FC with the ipsilesional precentral gyrus. For each cluster, changes in FC (ΔFC) correlated with FM‐UL improvement (ΔFM‐UL) and were compared between groups. *p < 0.05, **p < 0.001 before versus after intervention. CON, control group; FM‐UL, Fugl‐Meyer Assessment Upper Limb; IPL, inferior parietal lobule; MIT, motor imagery training group.

The two clusters mentioned above, showing significant *Group* × *Time* interaction and significant correlation with FM‐UL improvement, were selected as the seeds for the seed‐based FC analysis. Significantly different alterations in FC with the ipsilesional M1 (seed) were found between the two groups. Additionally, some FC changes correlated significantly with FM‐UL improvement (Figure 6 and Table S3). The ipsilesional inferior parietal lobule (IPL) (cluster size = 112, peak MNI: 30, −54, 48) and putamen (cluster size = 109, peak MNI: 34, −12, 18) had the highest concentration of FC with significant *Group* × *Time* interaction and correlation with FM‐UL improvement. Post hoc analysis (Figure 5A) revealed that FC between the ipsilesional IPL and the ipsilesional M1 decreased significantly in the MIT group after intervention (*p* < 0.001) compared with the CON group (*p* = 0.68). Moreover, the change in FC was significantly negatively correlated with FM‐UL improvement in all patients (*r* = −0.529, *p* = 0.003). In addition, FC with the ipsilesional M1 increased significantly in the ipsilesional putamen in the MIT group (*p* = 0.004) compared with the CON group (*p* = 0.113, Figure 5B). Meanwhile, the change in FC showed a significant positive correlation with FM‐UL improvement (*r* = 0.717, *p* < 0.001).

When taking the cluster in the contralesional S1 as the seed, no FC with both significant *Group* × *Time* interaction and correlation with FM‐UL improvement was found.

## 4 DISCUSSION

Emerging neuroimaging studies have identified neuroimaging biomarkers of functional recovery in stroke patients.^39^, ^40^, ^41^ Using multimodal neuroimaging approaches, recent discoveries have revealed brain responses to interventions targeting motor function, including brain atrophy alleviation,^42^ selective disruption of sensorimotor circuits,^43^, ^44^ different cortical recruitment patterns,^9^ and intrinsic brain network reorganization.^33^ Using task‐based fMRI, this longitudinal study explored the unique MIT‐related brain reorganization in stroke patients. The activation pattern and seed‐based FC examination were conducted when patients performed a passive finger flexion–extension task on both hands before and after 4 weeks of rehabilitation training. Our results suggested that MIT could help decrease the compensatory activation in the two hemispheres and reshape the FC within the ipsilesional hemisphere along with functional recovery in stroke patients.

The activation of the passive movement of the affected hand showed an abnormal bilateral activation pattern compared with the contralateral‐lateralized activation of the passive movement of the unaffected hand. When stroke patients passively moved the unaffected hand, the activation focused on the contralateral sensorimotor cortex (contralesional M1, S1, SMA) and ipsilateral (ipsilesional) cerebellum, presenting a typical activation pattern that was similar to cortical representation for hand motor tasks in healthy individuals.^45^ However, when stroke patients passively moved the affected hand, bilateral sensorimotor networks (mainly in SMA and cerebellum) were activated, indicating relocation of motor‐related regions across the two hemispheres providing temporary functional compensation after stroke. The findings are consistent with previous studies, which have shown that frontoparietal and cerebellar regions on ipsilesional and contralesional hemispheres were activated by active or passive hand movements in stroke patients.^28^, ^46^, ^47^ A longitudinal fMRI study using a passive wrist‐extension task found significant positive correlations between hand motor performances and bilateral sensorimotor activity in the early phase of chronic stroke, suggesting that the contralesional motor network contributes to functional repair.^47^ Additionally, a significant increase in activation in the motor areas of both hemispheres during hand movement has been observed in stroke patients with severe upper limb motor impairments.^48^ However, no significant difference in activation maps during passive hand movement was observed between stroke and healthy participants in well‐recovered patients.^47^ These seemingly contradictory results can be explained by the bimodal balance‐recovery model of stroke that increased fMRI activation in the bilateral brain hemispheres during hand movement tasks have dual effects in patients with different levels of motor recovery.^49^

After MIT, the compensatory activation of bilateral sensorimotor networks decreased. The decrease was associated with the adjunctive efficacy of MIT. The activation of the passive movement of the affected hand in the contralesional S1 and ipsilesional M1 decreased significantly after the intervention in the MIT group compared with the control group. Correlation analyses further demonstrated that decreased activation in these two areas was significantly negatively correlated with FM‐UL improvement. These findings suggest that the withdrawal of bilateral compensation was associated with better motor recovery in the MIT group. Previous studies have suggested that although the temporary compensatory activation could support the functional improvement in the acute and subacute phases after stroke, in the long run, overreliance on compensatory brain activation may hinder the recovery of motor ability.^50^ It has been demonstrated that decompensation of the contralesional hemisphere is required for better recovery after stroke. Patients with fast motor recovery showed strong deactivation in the contralesional sensorimotor area.^51^ Similar to our study, Liu et al.^20^ found that MIT can promote this decompensation process to re‐establish the normal contralateral‐lateralized pattern for stroke patients, which was associated with their hand functional recovery. According to the interhemispheric balance theory, the diminished compensatory activation in the contralesional sensorimotor area may contribute to motor output by inhibiting influences from the undamaged hemisphere after MIT. Comparatively, functional neuroanatomy can be used to explain the decreased activation in the ipsilesional M1. The peak MNI coordinate of the ipsilesional M1 with declined activation in the MIT group was (12, −24, 69), deviating from the motor hand area that peaked at the coordinate of (38, −22, 56) from a previous study.^52^ In our study, the decreased ipsilesional M1 was located medial to the hand motor area and overlapped the motor areas of the proximal part of the upper extremity.^53^ Before the intervention, the activation in this region was possibly abnormally increased as a temporary change to provide more resources for motor output in stroke patients with severe injury. MIT intervention facilitated the decrease of compensatory overactivity in ipsilesional M1, promoting the recovery of hand motor function in stroke patients.

Moreover, it was observed that the effects of MIT were found only on the brain activation of the affected hand and had no effect on the unaffected hand. The findings indicate that the training‐induced brain plasticity was restricted to BOLD responses of the MIT‐targeted hand since patients were only instructed to imagine moving the hand and upper limb at the affected side during the training. Likewise, TMS studies have revealed that the increase in cortico‐spinal excitability is specific to the muscles involved in the imagined movement.^54^ Imagined thumb opposition to the little finger, for example, only increased the MEP of opponens pollicis and the first dorsal interosseus dedicated to this movement. At the same time, the MEP of other intrinsic hand muscles, such as abductor digiti minimi, remained unchanged.^55^ In addition to the findings of targeted effects of MIT, the consistent activation pattern during passive movement of the unaffected hand before and after intervention in both groups demonstrated that the task‐evoked BOLD activity was a stable and robust paradigm that reflected the functional level of the corresponding limb.

MIT potential mechanisms for stroke treatment may also associate with the changes in FC with the ipsilesional M1. The FC of the ipsilesional M1 significantly decreased in the ipsilesional IPL and increased in the ipsilesional putamen after MIT but remained unchanged after sole CRT. The change in these FCs was significantly correlated with FM‐UL improvement. The change of FC patterns indicated that the remodeling of activation time course synchronization mainly occurred within the damaged hemisphere and reflected the possible neural substrate underlying the rehabilitation effects of MIT on the motor functions of the patient. Similarly, change in FC has been observed in a resting‐state fMRI study. Zhang et al.^56^ identified an abnormal increase in FC between the ipsilesional M1 and the ipsilesional IPL in stroke patients before the intervention, which disappeared after 30 days of MIT therapy. In a large cohort of 132 individuals with deficits affecting a range of post‐stroke dysfunctions (37 with the left motor deficit, 39 with the right motor deficit), Siegel et al.^57^ proposed a network phenotype of stroke injury. Their results indicated that while interhemispheric connectivity abnormally decreased, an abnormal increase in connectivity within the ipsilesional hemisphere was observed. Along with functional recovery after treatment, the FC between ipsilateral frontal and parietal regions decreased, while the FC between ipsilateral frontal regions and posterior parietal‐occipital‐temporal areas increased.^57^, ^58^

The IPL of the human brain is engaged in numerous mental processes such as visuospatial attention, memory, and mathematical cognition.^59^ Structure connectivity and resting‐state functional connectivity between IPL and premotor cortex have been found and are essential in transforming stimuli from the visual system to the motor system coordinating visuomotor actions such as reaching, grasping, and eye movements.^60^ Taken together, we speculate that the increased FC between ipsilesional IPL and M1 before intervention in our study could be part of a compensatory strategy that integrated visual system information to support the action output in patients with severe motor impairments. The brain motor network became more efficient with the recovery of motor function after MIT therapy. As a result, the compensatory support of IPL, which coordinated visual information into motor action, gradually lost its significance. This explains the negative correlation observed between upper limb motor improvement and the change in FC between ipsilesional M1 and IPL.

The enhanced FC resulting from MIT therapy might improve upper limb fine movements, creating a positive correlation between FM‐UL improvement and change in FC relative to the ipsilesional putamen. The lentiform nucleus comprises putamen and globus pallidus which combine with the caudate nucleus to shape the striatum. The striatum receives afferent input from different parts of the cortex and sends efferent output to the cortex through the thalamus.^61^ There are connections between the anterior portion of the putamen and associated regions exist in the cortex; the posterior portion of the putamen connects to the primary motor cortex and the supplementary motor area.^62^ Available evidence suggests that subcortical regions such as the putamen and thalamus are important regulators of fine motor rehabilitation with previously learned movements.^63^ Enhanced FCs from the putamen to primary motor regions has been reported in patients with Parkinson's disease^64^ and stroke.^65^ A resting‐state FC study revealed that the increased FC from the contralesional thalamus in patients with supratentorial stroke compared with healthy individuals and was correlated positively with motor improvement at 6‐month follow‐up.^66^ Besides static FC analysis, the dynamic approach of FC analysis found that the variability of the connectivity between the ipsilesional sensorimotor cortex and putamen could discriminate patients with different levels of motor function.^67^ Furthermore, during the process of motor imagery, the subcortical motor areas, like the basal ganglia, are recruited.^68^ According to a study of 37 hemiplegic stroke patients, impaired motor imagery capacity was associated with putamen damage.^69^ A recent systematic review also revealed that damage to putamen suppresses motor imagery capacities.^70^ Herein, most stroke lesions involved subcortical regions, including basal ganglia, centrum ovale, corona radiate, thalamus, and internal capsule. These subcortical lesions disrupt the anatomical connections between these areas and sensorimotor areas. Our findings revealed that the enhanced connection between ipsilesional M1 and ipsilesional putamen might regulate voluntary motor skills and motor relearning through motor imagery training after brain damage. The improvement of upper limb function in the MIT group is potentially related to the repair of this connection at the functional level; this suggests that the neural basis of MIT is reflected in brain activation and motor network remodeling.

The current study has several limitations that should be noted. Firstly, the FM‐UL was used as the only clinical assessment of upper limb motor function. Thus, additional comprehensive assessments, such as the Wolf Motor Function Test, Action Research Arm Test, and Box and Block Test, should be applied in future studies to improve the interpretation of results. Secondly, we calculated the sample size based on our preliminary data and related studies. However, only 29/39 patients with complete fMRI data were included in the analysis due to the dropout and excessive head motion during MRI scanning. Therefore, more participants should be recruited in the future to increase the validity of the results. Thirdly, our results indicated that the contralesional S1, the ipsilesional M1, IPL, and putamen were key sensorimotor nodes which correlated with upper limb motor recovery after MIT. Although these brain areas modulate motor planning, motor execution, and motor learning, it remains unclear whether these nodes participate in neuromodulation when combined with MIT. In the future, an intervention study combining MIT and noninvasive brain stimulation is advocated to promote the development of novel treatment paradigms to improve stroke recovery.

## 5 CONCLUSION

In conclusion, this is an inaugural study that uses task‐based fMRI to investigate the neural mechanism of MIT in stroke patients with moderate to severe upper limb motor impairments. This work expands our understanding of the effects of MIT on brain reorganization both at the activation and brain network levels. Besides, our findings demonstrate that MIT can decrease overaction at both hemispheres and reorganize the motor network within the ipsilesional hemisphere, thereby promoting upper limb motor function during stroke recovery.

## AUTHOR CONTRIBUTIONS

LMS and XLG were involved in the development and design of the study concept; CHS, BZ, and YMX were involved in intervention and assessment; XX, KXZ, XW, MXF, and SBT were involved in data acquisition and analysis; HWW and XX contributed to the initial manuscript writing. All authors revised and agreed to the final version of this article.

## CONFLICT OF INTEREST

All the authors declared no conflicts of interest.

## Supporting information


Table S1‐S3

## Data Availability

The data that support the findings of this study are available from the corresponding author upon reasonable request.
