# Supplementary material for: Motor network reorganization after motor imagery training in stroke patients with moderate to severe upper limb impairment
Source: CNS Neurosci Ther. 2022 Dec 27;29(2):619–32. doi: 10.1111/cns.14065 (PMC9873524; doi:10.1111/cns.14065)
Supplement: Supplementary file 1 — Table S1‐S3 [file CNS-29-619-s001.docx]

Supplementary Table S1. Brain activation to passive movements of the affected and unaffected hands in both groups before and after intervention.

| **Brain Region** | **Cluster Size (voxels)** | **PeakT_max_** | **MNI Coordinates** | | |
| --- | --- | --- | --- | --- | --- |
|  |  |  | **x** | **y** | **z** |
| **Activation to passive movement of the unaffected hand** | | | | | |
| **CON group before intervention** | | | | | |
| Postcentral gyrus_ contra | 1616 | 9.24 | -48 | -21 | 30 |
| Precentral gyrus_ contra | 297 |  | | | |
| Postcentral gyrus_ contra | 267 |  | | | |
| Supplementary motor area_ contra | 247 |  | | | |
| Superior temporal gyrus_ contra | 207 |  | | | |
| Median cingulate and paracingulate gyri_ contra | 95 |  | | | |
| Supramarginal gyrus_ contra | 81 |  | | | |
| Supplementary motor area_ ipsi | 75 |  | | | |
| Cerebellum_ ipsi | 240 | 5.71 | 9 | -48 | -15 |
| Putamen_ contra | 178 | 5.69 | -15 | -21 | 9 |
| **CON group after intervention** | | | | | |
| Precentral gyrus_ contra | 260 | 5.43 | -36 | -18 | 54 |
| Postcentral gyrus_ contra | 132 |  | | | |
| Precentral gyrus_ contra | 121 |  | | | |
| Cerebellum_ipsi | 164 | 4.91 | 21 | -57 | -21 |
| Supplementary motor area_ contra | 151 | 5.13 | -3 | -9 | 60 |
| Superior temporal gyrus_ contra | 104 | 5.75 | -39 | -30 | 15 |
| **MIT group before intervention** | | | | | |
| Postcentral gyrus_ contra | 2441 | 16.35 | -39 | -27 | 48 |
| Postcentral gyrus_ contra | 614 |  | | | |
| Precentral gyrus_ contra | 432 |  | | | |
| Supplementary motor area_ contra | 253 |  | | | |
| Superior temporal gyrus_ contra | 214 |  | | | |
| Supramarginal gyrus_ contra | 174 |  | | | |
| Inferior parietal lobule_ contra | 117 |  | | | |
| Rolandic operculum_ contra | 106 |  | | | |
| Median cingulate and paracingulate gyri_ contra | 98 |  | | | |
| Superior parietal gyrus_ contra | 76 |  | | | |
| Supplementary motor area_ ipsi | 70 |  | | | |
| Cerebelum_ ipsi | 790 | 10.98 | 15 | -54 | -15 |
| Cerebelum_ ipsi | 248 | 9.22 | 15 | -63 | -48 |
| Thalamus_ contra | 157 | 7.38 | -27 | -21 | 9 |
| **MIT group after intervention** | | | | | |
| Postcentral gyrus_ contra | 1441 | 10.48 | -39 | -30 | 51 |
| Postcentral gyrus_ contra | 540 |  | | | |
| Precentral gyrus_ contra | 272 |  | | | |
| Supramarginal gyrus_ contra | 132 |  | | | |
| Supplementary motor area_ contra | 127 |  | | | |
| Inferior parietal lobule_ contra | 113 |  | | | |
| Cerebelum_ ipsi | 316 | 6.55 | 18 | -54 | -18 |
| **Activation to passive movement of the affected hand** | | | | | |
| **CON group before intervention** | | | | | |
| Vermis | 233 | 5.75 | 3 | -72 | -12 |
| Cerebelum_ contra | 118 |  | | | |
| Cerebelum_ ipsi | 56 |  | | | |
| Supplementary motor area_ ipsi | 206 | 5.04 | 12 | -3 | 63 |
| Supplementary motor area_ ipsi | 101 |  | | | |
| Supplementary motor area_ contra | 91 |  | | | |
| Supramarginal gyrus_ ipsi | 180 | 6.03 | 54 | -33 | 30 |
| Superior temporal gyrus_ contra | 110 | 5.48 | -69 | -21 | 9 |
| **CON group after intervention** | | | | | |
| Superior temporal gyrus_ contra | 679 | 8.11 | -66 | -12 | 6 |
| Superior temporal gyrus_ contra | 353 |  | | | |
| Supramarginal gyrus_ contra | 70 |  | | | |
| Supplementary motor area_ ipsi | 583 | 8.02 | 12 | 9 | 54 |
| Supplementary motor area_ ipsi | 235 |  | | | |
| Supplementary motor area_ contra | 190 |  | | | |
| Superior temporal gyrus_ ipsi | 163 | 5.73 | 51 | -27 | 6 |
| **MIT group before intervention** | | | | | |
| Supplementary motor area_ ipsi | 1777 | 12.3 | 6 | -3 | 54 |
| Supplementary motor area_ ipsi | 344 |  | | | |
| Postcentral gyrus_ ipsi | 342 |  | | | |
| Supplementary motor area_ contra | 304 |  | | | |
| Precentral gyrus_ contra | 246 |  | | | |
| Median cingulate and paracingulate gyri_ ipsi | 137 |  | | | |
| Median cingulate and paracingulate gyri_ contra | 126 |  | | | |
| Supramarginal gyrus_ contra | 918 | 10.53 | -54 | -36 | 24 |
| Superior temporal gyrus_ contra | 314 |  | | | |
| Supramarginal gyrus_ contra | 259 |  | | | |
| Postcentral gyrus_ contra | 131 |  | | | |
| Rolandic operculum_ contra | 906 | 5.02 | -48 | 0 | 12 |
| Precentral gyrus_ contra | 294 |  | | | |
| Inferior frontal gyrus, opercular part_ contra | 185 |  | | | |
| Rolandic operculum_ contra | 98 |  | | | |
| Insula_ contra | 88 |  | | | |
| Cerebelum_ ipsi | 255 | 7.55 | 27 | -54 | -24 |
| Cerebelum_ contra | 112 | 6.10 | -27 | -60 | -48 |
| **MIT group after intervention** | | | | | |
| Postcentral gyrus_ ipsi | 252 | 7.26 | 42 | -36 | 66 |
| Postcentral gyrus_ ipsi | 141 |  | | | |
| Precentral gyrus_ ipsi | 106 |  | | | |
| Superior temporal gyrus_ contra | 159 | 4.90 | -60 | -36 | 18 |
| Supramarginal gyrus_ contra | 77 |  | | | |
| Superior temporal gyrus_ contra | 66 |  |  |  |  |

All clusters are significant at voxel-level uncorrected p < 0.001 and cluster size ≥ 100 voxels. Regions are reported using Automated Anatomical Labelling (AAL) template and clusters that across multiple regions are listed in detail (region size > 50 voxels). MIT, motor imagery training group; CON, control group; contra, contralesional; ipsi, ipsilesional.

Supplementary Table S2. Different alterations in brain activation to passive movement of the affected hand between the motor imagery training group (MIT) and control group (CON).

| **Brain Region** | **Cluster Size (voxels)** | **PeakT_max_** | **MNI Coordinates** | | |
| --- | --- | --- | --- | --- | --- |
|  |  |  | **x** | **y** | **z** |
| **Activation with significant *Group* × *Time* interaction** | | | | | |
| Postcentral gyrus_ contra | 298 | 3.30 | -60 | -30 | 48 |
| Postcentral gyrus_ contra | 94 |  | | | |
| Precentral gyrus_ contra | 71 |  | | | |
| Paracentral lobule_ ipsi | 105 | 3.26 | 12 | -24 | 69 |
| Precentral gyrus_ ipsi | 61 |  |  |  |  |
| **Activation change positively correlates with FM-UL improvement** | | | | | |
| Cerebelum_ ipsi | 198 | 1.72 | 15 | -51 | -42 |
| Precuneus_ contra | 114 | 2.16 | 0 | -45 | 15 |
| **Activation change negatively correlates with FM-UL improvement** | | | | | |
| Inferior frontal gyrus, opercular part_ contra | 6235 | 4.81 | -57 | 15 | 33 |
| Postcentral gyrus_ contra | 643 |  | | | |
| Precentral gyrus_ contra | 555 |  | | | |
| Superior temporal gyrus_ contra | 367 |  | | | |
| Inferior parietal lobule_ contra | 319 |  | | | |
| Supplementary motor area_ ipsi | 317 |  | | | |
| Inferior frontal gyrus, triangular part_ contra | 310 |  | | | |
| Supplementary motor area_ contra | 301 |  | | | |
| Postcentral gyrus_ ipsi | 258 |  | | | |
| Supramarginal gyrus_ contra | 220 |  | | | |
| Middle temporal gyrus_ contra | 217 |  | | | |
| Precentral gyrus_ ipsi | 213 |  | | | |
| Superior parietal gyrus_ ipsi | 196 |  | | | |
| Inferior frontal gyrus, opercular part_ contra | 155 |  | | | |
| Paracentral lobule_ ipsi | 132 |  | | | |
| Superior frontal gyrus_ ipsi | 127 |  | | | |
| Inferior occipital gyrus_ ipsi | 423 | 3.34 | 45 | -63 | -15 |
| Superior temporal gyrus_ ipsi | 112 |  |  |  |  |
| Middle temporal gyrus_ ipsi | 102 |  |  |  |  |
| Inferior frontal gyrus_ ipsi | 301 | 4.31 | 36 | 33 | -9 |
| **Activation with significant *Group* × *Time* interaction & correlation with FM-UL improvement** | | | | | |
| Postcentral gyrus_ contra | 184 | 3.30 | -59 | -29 | 47 |
| Precentral gyrus_ ipsi | 100 | 3.25 | 12 | -24 | 69 |

All clusters are significant at threshold-free cluster enhancement [TFCE] corrected p < 0.05 and cluster size ≥ 100 voxels. Regions are reported using Automated Anatomical Labelling (AAL) template and clusters that across multiple regions are listed in detail (region size > 50 voxels). Contra, contralesional; ipsi, ipsilesional.

Supplementary Table S3. Different alterations in functional connectivity with the ipsilesional SMA&M1 between the motor imagery training group (MIT) and control group (CON).

| **Brain Region** | **Cluster Size (voxels)** | **PeakT_max_** | **MNI Coordinates** | | |
| --- | --- | --- | --- | --- | --- |
|  |  |  | **x** | **y** | **z** |
| **FC with significant *Group* × *Time* interaction** | | | | | |
| Superior temporal gyrus_ ipsi | 4177 | 5.03 | 42 | -30 | 8 |
| Middle frontal gyrus_ contra | 1557 | 4.14 | -42 | 26 | 6 |
| Supplementary motor area_ contra | 975 | 4.12 | -14 | 18 | 68 |
| Superior frontal gyrus_ ipsi | 432 | 3.79 | 24 | -4 | 68 |
| Middle frontal gyrus_ contra | 235 | 3.28 | -44 | 18 | 36 |
| Cingulate | 127 | 3.22 | -12 | -24 | 32 |
| Putamen_ ipsi | 92 | 3.21 | 22 | 20 | 0 |
| **FC with significant *Group* × *Time* interaction** | | | | | |
| Middle occipital gyrus_ ipsi | 708 | 3.31 | 12 | -98 | 24 |
| Middle temporal gyrus_ contra | 391 | 3.92 | -38 | -10 | -50 |
| Temporal pole: middle temporal gyrus_ ipsi | 271 | 3.20 | 36 | 24 | -44 |
| Inferior parietal lobule_ ipsi | 245 | 3.63 | 30 | -54 | 48 |
| Middle frontal gyrus, opercular part_ ipsi | 218 | 3.91 | 0 | 48 | -10 |
| Inferior temporal gyrus_ ipsi | 178 | 3.50 | 48 | -20 | -40 |
| Cerebelum_ ipsi | 151 | 4.31 | 24 | -84 | -16 |
| **FC change positively correlates with FM-UL improvement** | | | | | |
| Inferior parietal lobule_ contra | 934 | 4.47 | -44 | -48 | 62 |
| Superior frontal gyrus, medial_ contra | 604 | 3.15 | -8 | 42 | 50 |
| Supramarginal gyrus_ ipsi | 263 | 2.63 | 66 | -28 | 26 |
| Putamen_ ipsi | 212 | 4.55 | 24 | -6 | 20 |
| Precuneus_ contra | 162 | 4.72 | -6 | -64 | 62 |
| Cerebelum_ ipsi | 161 | 5.38 | 12 | -54 | -58 |
| Superior frontal gyrus, medial_ contra | 100 | 2.01 | 0 | 62 | 20 |
| Paracentral lobule_ contra | 100 | 4.23 | -8 | -40 | 78 |
| **FC change negatively correlates with FM-UL improvement** | | | | | |
| Superior parietal gyrus_ ipsi | 738 | 4.45 | 30 | -60 | 50 |
| Precuneus_ ipsi | 224 | 2.98 | 6 | -42 | 56 |
| Middle occipital gyrus_ contra | 154 | 3.39 | -32 | -72 | 18 |
| Inferior frontal gyrus, triangular part_ ipsi | 141 | 1.65 | 52 | 26 | 6 |
| Cuneus_ contra | 105 | 2.77 | -2 | -88 | 30 |
| **FC with significant *Group* × *Time* interaction & correlation with FM-UL improvement** | | | | | |
| Inferior parietal lobule _ ipsi | 112 | 3.63 | 30 | -54 | 48 |
| Putamen_ ipsi | 109 | 3.58 | 34 | -12 | 18 |

All clusters are significant at threshold-free cluster enhancement [TFCE] corrected p < 0.05 and cluster size ≥ 100 voxels. Regions are reported using Automated Anatomical Labelling (AAL) template. Contra, contralesional; ipsi, ipsilesional.
